# Эпидемиология доброкачественных заболеваний щитовидной железы у взрослого населения Республики Беларусь: анализ общенациональных статистических данных за период 2009–2019 гг.

**DOI:** 10.14341/probl12844

**Published:** 2022-03-08

**Authors:** С. В. Якубовский, Г. Г. Кондратенко, О. Б. Салко, Е. И. Кузьменкова

**Affiliations:** Белорусский государственный медицинский университет; Белорусский государственный медицинский университет; Республиканский центр медицинской реабилитации и бальнеолечения; Республиканский центр медицинской реабилитации и бальнеолечения

**Keywords:** щитовидная железа, эпидемиология, зоб, гипотиреоз, тиреотоксикоз, тиреоидит

## Abstract

**АКТУАЛЬНОСТЬ:**

АКТУАЛЬНОСТЬ. В настоящее время Республика Беларусь (РБ) относится к странам с адекватной йодной обеспеченностью, что позволило снизить заболеваемость нетоксическим зобом и врожденным гипотиреозом. Однако даже незначительное изменение потребления йода разнонаправленно меняет характер наблюдаемой тиреоидной патологии. Помимо йододефицита, в этиопатогенезе заболеваний щитовидной железы (ЩЖ) значимую роль играют и другие условия окружающей среды, а также генетические факторы.

**ЦЕЛЬ:**

ЦЕЛЬ. Проанализировать динамику основных эпидемиологических показателей доброкачественных заболеваний ЩЖ с 2009 по 2019 гг. у взрослого населения РБ, используя данные официальной государственной статистики.

**МАТЕРИАЛЫ И МЕТОДЫ:**

МАТЕРИАЛЫ И МЕТОДЫ. Изучены показатели заболеваемости и распространенности доброкачественных заболеваний ЩЖ на основании государственных статистических данных за 2009–2019 гг. Для анализа динамики изучаемых показателей использован регрессионный анализ с построением линейных и полиномиальных моделей.

**РЕЗУЛЬТАТЫ:**

РЕЗУЛЬТАТЫ. Выявлены снижение заболеваемости и распространенности диффузного эутиреоидного зоба и рост заболеваемости и распространенности узлового эутиреоидного зоба, тиреоидита, приобретенного гипотиреоза, болезни Грейвса, а также заболеваемости узловым токсическим зобом.

**ЗАКЛЮЧЕНИЕ:**

ЗАКЛЮЧЕНИЕ. Полученные данные свидетельствуют о росте распространенности большинства изученных нозологических форм, несмотря на адекватную йодную обеспеченность. Вышеуказанное обосновывает необходимость дальнейшего изучения причин возникновения выявленных тенденций, а также целесообразность разработки новых методов диагностики и лечения тиреоидной патологии.

## Abstract (en)

**BACKGROUND:**

BACKGROUND. Nowadays, the Republic of Belarus belongs to the countries with sufficient iodine supply, which made it possible to reduce the incidence of non-toxic goiter and congenital hypothyroidism. However, even a slight change in iodine consumption influences the pattern of thyroid diseases. In addition to iodine deficiency, other environmental conditions, as well as genetic factors, play a significant role in the etiology of thyroid diseases.

**AIM:**

AIM. To analyze the dynamics of the main epidemiological indicators of benign thyroid diseases from 2009 to 2019 in the adult population of the Republic of Belarus, using the data of official state statistics.

**MATERIALS AND METHODS:**

MATERIALS AND METHODS. The indicators of the incidence and prevalence of benign thyroid diseases were studied on the basis of state statistics for 2009–2019. To analyze the dynamics of the studied indicators, regression analysis was used with the construction of linear and polynomial models.

**RESULTS:**

RESULTS. A decrease in the incidence and prevalence of diffuse euthyroid goiter and an increase in the incidence and prevalence of nodular euthyroid goiter, thyroiditis, acquired hypothyroidism, Graves’ disease, as well as the incidence of nodular toxic goiter were revealed.

**CONCLUSION:**

CONCLUSION. Obtained data indicate, that there is an increase in the prevalence of most of the studied thyroid diseases, despite the adequate iodine supply. The above justifies the need for further study of the causes of the identified trends, as well as the necessity of developing new methods of diagnosis and treatment of thyroid diseases.

## ВВЕДЕНИЕ

В настоящее время патология щитовидной железы (ЩЖ), наряду с сахарным диабетом, занимает лидирующие позиции по частоте встречаемости в структуре эндокринных заболеваний [1]. Спектр заболеваний ЩЖ чрезвычайно широк и варьирует от минимальных структурных изменений, не влияющих на жизнь пациентов, до существенных функциональных и морфологических расстройств, снижающих качество, а в ряде случаев и длительность жизни пациентов.

Возникновение заболеваний ЩЖ объясняется множеством факторов, как эндогенных, так и экзогенных. К наиболее изученным факторам окружающей среды относится потребление йода [2][3].

Длительный йодный дефицит приводит к формированию таких йододефицитных заболеваний, как диффузный и узловой/многоузловой зоб, тиреотоксикоз на фоне узловой патологии ЩЖ, врожденный гипотиреоз, умственная и физическая отсталость детей, кретинизм, невынашивание беременности; существенно увеличивается риск возникновения радиационно-индуцированного рака ЩЖ в случае атомных аварий. Для территорий с достаточным или избыточным потреблением йода в большей степени характерно развитие аутоиммунной патологии ЩЖ (аутоиммунный тиреоидит и болезнь Грейвса), а также диффузного зоба при избытке йода [3].

Исторически территория Республики Беларусь (РБ) являлась йододефицитной территорией. После внедрения в 2000 г. программы по ликвидации йодного дефицита, проводимой в соответствии с постановлением Главного санитарного врача №11 от 21.03.2000 «О предупреждении заболеваний, связанных с дефицитом йода», и одноименным Постановлением Совета Министров №484 от 06.04.2001, а также Законом Республики Беларусь «О качестве и безопасности продовольственного сырья и пищевых продуктов для жизни и здоровья человека» от 29.06.2003, РБ относится к странам с адекватной йодной обеспеченностью, что было ранее неоднократно продемонстрировано в ходе ряда исследований, оценивавших концентрацию йода в моче у репрезентативных групп населения [4][5]. Ликвидация йодного дефицита позволила снизить заболеваемость нетоксическим зобом и врожденным гипотиреозом [6].

Однако даже незначительное увеличение потребления йода в ранее йододефицитных популяциях разнонаправленно меняет характер наблюдаемой тиреоидной патологии [3]. С одной стороны, снижается заболеваемость диффузным и узловым зобом, врожденным гипотиреозом и иными йододефицитными заболеваниями и растет заболеваемость аутоиммунной патологией ЩЖ. С другой стороны, ряд изменений структуры и функции ЩЖ сохраняются, несмотря на увеличение потребления йода, что отражает не только текущий, но и предшествующий статус йодной обеспеченности (эффект «йодной памяти») [7].

Таким образом, при изучении эпидемиологии заболеваний ЩЖ необходимо принимать во внимание не только текущую йодную обеспеченность, но и историю потребления йода в конкретной популяции. Помимо этого, значительную роль играют и другие условия окружающей среды (уровень обеспеченности иными микронутриентами, воздействие ионизирующей радиации и т.д.), а также генетические факторы [3][6].

## ЦЕЛЬ ИССЛЕДОВАНИЯ

Проанализировать динамику основных эпидемиологических показателей доброкачественных заболеваний ЩЖ с 2009 по 2019 гг. у взрослого населения РБ, используя данные официальной государственной статистики.

## МАТЕРИАЛЫ И МЕТОДЫ

На основании статистических данных Государственного учреждения «Республиканский научно-практический центр медицинских технологий, информатизации, управления и экономики здравоохранения» за 2009–2020 гг. изучены показатели заболеваемости (отношение абсолютного числа случаев впервые выявленного заболевания к численности населения, умноженное на 100 000 человек), кумулятивной заболеваемости (отношение абсолютного числа случаев впервые выявленного заболевания, возникших за определенный период времени, к численности населения, умноженное на 100 000 человек в начале этого периода времени) и распространенности (отношение абсолютного числа случаев заболевания к численности населения, умноженное на 100 000 человек) доброкачественных заболеваний ЩЖ у взрослого населения РБ.

Заболевания были разделены по группам в соответствии с используемой в статистической отчетности структурой, базирующейся на международной классификации болезней 10-го пересмотра: приобретенный гипотиреоз (Е02–03), нетоксический диффузный зоб (Е04.0), нетоксический одноузловой и многоузловой зоб (Е04.1–Е04.2), тиреотоксикоз (гипертиреоз) с токсическим одноузловым и многоузловым зобом (Е05.1–Е05.2), тиреоидит (Е06), тиреотоксикоз с диффузным зобом (Е05.0)

В ходе выполнения работы было выявлено, что 2020 г. характеризовался нетипичными изменениями в показателях заболеваемости и распространенности ряда нозологий, что может быть объяснено возникновением пандемии SARS-CoV-2. В связи с этим данные за 2020 г. были исключены из анализа.

Для анализа динамики изучаемых показателей использован регрессионный анализ с построением линейных и полиномиальных моделей. Выбор типа модели основывался на показателе коэффициента детерминации R2, отражающем соответствие аппроксимирующей кривой исходным данным. Для линейной регрессии рассчитывался угол наклона линии регрессии (коэффициент регрессии b), позволяющий количественно отразить динамику процесса. Для оценки статистической значимости угла наклона применялся t-критерий Стьюдента: наличие динамики считалось статистически значимым при p<0,05 [8]. Обработка данных проводилась с использованием пакета программ Microsoft Excel 2019 (построение линейных и полиномиальных моделей, коэффициент детерминации) и STATISTICA 10 (коэффициент регрессии b, t-критерий Стьюдента).

## ЭТИЧЕСКАЯ ЭКСПЕРТИЗА

Исследование одобрено Комитетом по биомедицинской этике Учреждения образования «Белорусский государственный медицинский университет» Министерства здравоохранения РБ (протокол №4 от 01 ноября 2021 г.).

## РЕЗУЛЬТАТЫ И ОБСУЖДЕНИЕ

Зоб и узлы щитовидной железы

Наиболее частыми патологиями ЩЖ являются диффузный и узловой/многоузловой зоб; их распространенность в значительной степени определяется содержанием йода в окружающей среде, поэтому эти заболевания традиционно используются для оценки йодной обеспеченности [9].

Взаимосвязь между потреблением йода и риском развития диффузного зоба имеет форму U-образной кривой: и недостаток, и избыток йода сопровождаются ростом заболеваемости диффузным зобом. Риск возникновения узлового зоба увеличивается при недостатке йода [3]. В частности, в ходе проспективного исследования, проведенного в трех провинциях Китая с различной йодной обеспеченностью (недостаточной — медианная концентрация йода в моче 88 мкг/л, достаточной — 214 мкг/л и избыточной — 634 мкг/л), было установлено, что кумулятивная заболеваемость за 5-летний период диффузным зобом составила 7100, 4400 и 6900 случаев, а узловым зобом — 5000, 2400 и 800 случаев на 100 тыс. населения соответственно [10].

Было изучено влияние нормализации йодной обеспеченности на динамику показателей заболеваемости диффузным и узловым зобом. В Дании через 4 года после внедрения программы йодирования соли в йододефицитных регионах было отмечено уменьшение среднего объема ЩЖ [11], а через 11 лет — уменьшение распространенности узлового зоба в сопоставимых возрастных группах, несмотря на сохранение распространенности узлового (5100 случаев на 100 000 населения) и рост распространенности многоузлового зоба (13 800 случаев на 100 000 населения), который наблюдался в регионе с предшествующим дефицитом йода средней тяжести [12]. В исследовании, выполненном по результатам ликвидации йодного дефицита в Италии, было установлено, что распространенность зоба снизилась прежде всего за счет диффузного зоба (с 34 000 до 10 300 случаев на 100 000 населения), в то время как распространенность узлового зоба снизилась с 11 300 до 3800 случаев на 100 000 населения только у лиц младше 35 лет; у лиц старше 35 лет она не изменилась [13].

Таким образом, коррекция йодной недостаточности у взрослого населения, независимо от тяжести, уменьшает средний размер ЩЖ и распространенность диффузного зоба во всех возрастных группах в течение нескольких лет. Что касается узлового зоба, восполнение йодного дефицита уменьшает риск возникновения узлов ЩЖ у молодых лиц, но не влияет на распространенность узлового зоба у лиц старших возрастных групп.

На территории РБ первичная заболеваемость диффузным эутиреоидным зобом снизилась у взрослых с 37,4 на 100 000 населения в 2009 г. до 26,2 на 100 000 населения в 2019 г. (рис. 1). Проведенный анализ показал статистически значимое снижение заболеваемости указанной патологией: b=-0,748 при р=0,020; R2=0,469. Кумулятивная заболеваемость за указанный период времени составила 314,35 на 100 000 населения.

Распространенность зоба за этот же период времени статистически значимо снизилась с 423,6 до 196,8 на 100 000 населения (b=-20,8 при р=0,000004; R2=0,916) (рис. 2).

Первичная заболеваемость узловым эутиреоидным зобом выросла у взрослых с 101,8 на 100 000 населения в 2009 г. до 168,4 на 100 000 населения в 2019 г. (рис. 3). Установлен статистически значимый рост заболеваемости указанной патологией: b=6,4 при р=0,000003; R2=0,923. Кумулятивная заболеваемость за указанный период времени составила 1370,96 на 100 000 населения.

Распространенность узлового эутиреоидного зоба за этот же период времени выросла с 1197,9 до 1756,3 на 100 000 населения (рис. 4). Выявлен статистически значимый рост распространенности указанной патологии: b=55,0 при р=0,000000005; R2=0,981.

**Figure:**
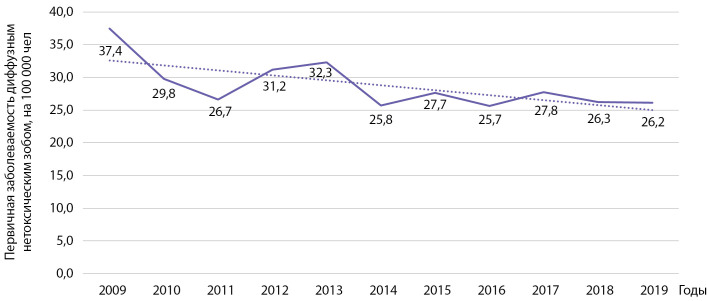
Рисунок 1. Динамика заболеваемости диффузным нетоксическим зобом на 100 000 человек.

**Figure:**
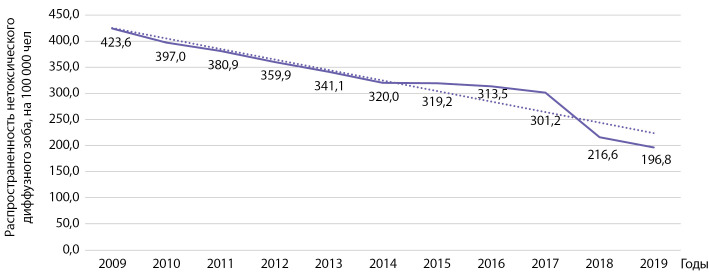
Рисунок 2. Динамика распространенности диффузного нетоксического зоба на 100 000 человек.

**Figure:**
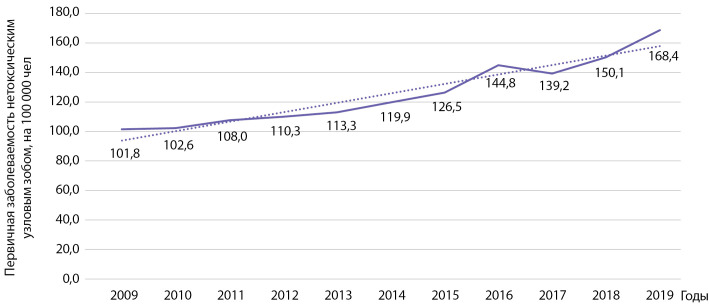
Рисунок 3. Динамика заболеваемости нетоксическим узловым зобом на 100 000 человек.

**Figure:**
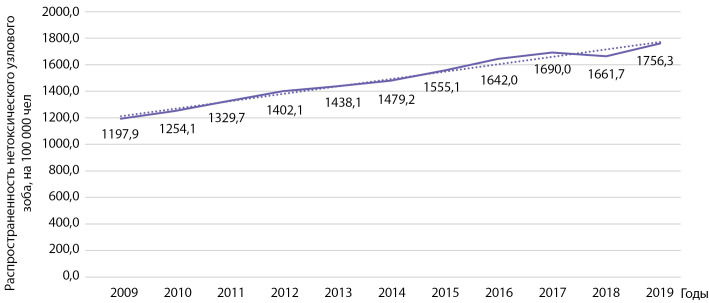
Рисунок 4. Динамика распространенности нетоксического узлового зоба на 100 000 человек.

Отмечаемая устойчивая тенденция к снижению показателей заболеваемости и распространенности диффузного зоба в определенной степени может объясняться достаточной йодной обеспеченностью; в случае узлового зоба отмечается достоверный рост этих показателей. Последнее, на наш взгляд, скорее всего, отражает общемировую тенденцию к увеличению разрешающей способности и доступности ультразвукового исследования ЩЖ и, соответственно, к выявлению большего количества узловых образований, зачастую не имеющих клинических проявлений [14]. Кроме того, существующие стандартные формы статистической отчетности не позволяют в полной мере изучить динамику заболеваемости в различных возрастных группах, что затрудняет более детальную оценку эффективности программы йодной профилактики возникновения узлового зоба.

Сопоставление наших данных с ранее опубликованными сведениями показывает, что текущая распространенность и заболеваемость населения РБ диффузным зобом ниже ранее опубликованных в исследованиях, проведенных в популяциях, проживающих на территориях с достаточной йодной обеспеченностью. Это может быть обусловлено как тем фактом, что материалы официальной статистики не всегда отражают истинную распространенность эндокринных заболеваний, поскольку частота регистрации заболеваний по обращаемости может быть в разы ниже частоты регистрации заболеваний по медицинским осмотрам [15], так и несопоставимостью результатов различных исследований вследствие отличия используемых диагностических критериев диффузного зоба [2]. Решение этой проблемы большинство исследователей видят в стандартизированном изучении объема ЩЖ при помощи ультразвукового исследования в ходе проспективных эпидемиологических исследований, что дает возможность выяснить истинную распространенность этой патологии и делает возможным сопоставление данных с результатами других исследований [2][6].

Узловые образования могут быть обнаружены у 60–70% представителей популяции [16]. В последние годы отмечается рост распространенности узлового зоба, что связывают не только с совершенствованием методов лучевой диагностики, но и с влиянием факторов окружающей среды, изменением образа жизни, нарушением процессов обмена веществ (ожирение, метаболический синдром) [17]. Поэтому истинная распространенность узлового зоба в популяции, несомненно, гораздо выше. Однако подавляющее число этих узловых образований не имеет клинической симптоматики и обычно не проявляет себя на протяжении всей жизни человека. Показаниями к хирургическому лечению узловых образований являются функциональная автономия, наличие компрессионного синдрома, косметический дефект, а также рак ЩЖ [18]. Основной проблемой, связанной с выявлением большого количества узловых образований, является дифференциальная диагностика морфологической природы этих узловых образований, которая пока позволяет установить характер узлов лишь в 70–80% случаев [19]. Это обусловливает необходимость совершенствования методов и протоколов диагностических исследований для более точной и надежной морфологической верификации диагноза на дооперационном этапе в условиях постоянно увеличивающегося потока пациентов.

Тиреоидит и приобретенный гипотиреоз

Понятие «тиреоидит» включает в себя различные формы заболевания: острый, подострый, аутоиммунный и другие более редкие формы. Превалирующей нозологической формой в этой группе является аутоиммунный тиреоидит (АИТ), который является основной причиной приобретенного гипотиреоза у взрослых; заболеваемость АИТ составляет 30–150 случаев на 100 000 человек в год [20].

В Европе распространенность гипотиреоза составляет в среднем 3050 (710–5050) случаев на 100 000 населения; заболеваемость — 226 случаев на 100 000 населения в год, варьируя от 13,5 до 297 случаев [21]. В России в 2018 г. распространенность гипотиреоза составила 446 случаев, а заболеваемость — 59 на 100 000 населения в год. Распространенность и заболеваемость тиреоидитом составили соответственно 428 и 48 случаев на 100 000 населения в год [22].

Для распространенности гипотиреоза и аутоиммунной патологии ЩЖ также характерен U-образный тип зависимости от количества потребляемого йода. В популяциях с тяжелой йодной недостаточностью распространенность гипотиреоза и частота повышения уровня тиреоидных антител выше, чем при оптимальном поступлении йода в организм [23][24]. Однако в условиях умеренного йодного дефицита распространенность субклинического и манифестного гипотиреоза меньше, чем на территориях с достаточным либо избыточным потреблением йода. При сравнении населения Дании, находившегося в условиях дефицита йода, и Исландии, для которого был характерен избыток йода, распространенность гипотиреоза была меньше в Дании, чем в Исландии, — 3800 и 18 000 случаев на 100 000 населения соответственно [25]. В ходе проспективного исследования, выполненного в Китае, была изучена заболеваемость гипотиреозом в когортах с дефицитом йода, оптимальной и избыточной йодной обеспеченностью. Было установлено, что, хотя кумулятивная заболеваемость манифестным гипотиреозом значимо не отличалась (200, 500 и 300 случаев на 100 000 населения соответственно), заболеваемость субклиническим гипотиреозом была достоверно выше при достаточном и избыточном потреблении йода (200, 2600, и 2900 случаев на 100 000 населения соответственно) [26].

Внезапное увеличение потребления йода, особенно при предшествующем его выраженном дефиците, сопровождается развитием либо усилением ранее протекавших аутоиммунных процессов в ткани ЩЖ, что может сопровождаться развитием субклинического или манифестного гипотиреоза. При сопоставлении 2 когорт датчан с различной степенью йодного дефицита заболеваемость манифестным гипотиреозом составила 29,7 случая на 100 000 населения в год в группе с более выраженным йодным дефицитом и 51,6 случая на 100 000 населения в год при легкой степени йодного дефицита. Через 7 лет после начала программы йодной фортификации было установлено, что в регионе с предшествующим йодным дефицитом средней тяжести (медианная концентрация йода в моче 45 и 86 мкг/л соответственно) заболеваемость манифестным гипотиреозом выросла и составила 40,3 случая на 100 000 населения в год, а в регионе с предшествующим легким йодным дефицитом (медианная концентрация йода в моче 61 и 99 мкг/л соответственно) заболеваемость манифестным гипотиреозом достоверно не изменилась и составила 56,7 случая на 100 000 населения в год [27]. После начала программы йодной фортификации в Италии также было выявлено увеличение распространенности гипотиреоза с 2800 до 5000 случаев на 100 000 населения, прежде всего за счет субклинического гипотиреоза у лиц моложе 15 лет [13].

Существует несколько возможных объяснений развития гипотиреоза на фоне избытка йода. Известно, что повышение содержания йода может приводить к повышению уровня тиреотропного гормона (ТТГ), что было доказано в эксперименте [28]. Это может объяснять тот факт, что у многих лиц, проживающих в условиях достаточного или избыточного поступления йода, обнаруживается повышение уровня сывороточного ТТГ и могут иметь место диффузные изменения при ультразвуковом исследовании, но при этом у них отсутствует повышение содержания антитиреоидных антител.

С другой стороны, быстрый рост потребления йода может стимулировать аутоиммунные процессы в ЩЖ за счет изменения антигенности тиреоглобулина и приводить к манифестации АИТ [29] либо оказывать прямое повреждающее действие на тиреоциты вследствие токсических эффектов йода и синтеза свободных радикалов [30][31].

В ряде исследований была продемонстрирована взаимосвязь между поступлением йода и развитием аутоиммунных процессов в ткани ЩЖ. При обследовании населения Дании до и через 4–5 лет после начала программы йодной фортификации было установлено увеличение частоты выявления повышенного уровня антител к ткани ЩЖ и субклинического гипотиреоза [32]. Схожие данные были получены в Италии в ходе 15-летнего наблюдения за лицами, включенными в программу йодной фортификации (частота АИТ выросла с 3500 до 14 500 случаев на 100 000 населения) [13]. При изучении на протяжении 5 лет влияния йодной обеспеченности на заболеваемость АИТ в Китае кумулятивная заболеваемость составила 200 случаев в условиях йодного дефицита, 1000 случаев — при достаточном йодном обеспечении и 1300 случаев на 100 000 населения — в условиях избытка йода [26].

Вместе с тем постепенное увеличение потребления йода, особенно на фоне его незначительного дефицита, не сопровождается значимым ростом заболеваемости аутоиммунной патологией [33].

Изучение долгосрочных результатов применения программ йодной фортификации показало, что через 15–20 лет после достижения достаточной йодной обеспеченности может отмечаться уменьшение распространенности гипотиреоза, АИТ и частоты повышения антител к ткани ЩЖ [34–36].

Таким образом, выраженность и частота развития аутоиммунных процессов в ткани ЩЖ, зачастую определяемые по содержанию тиреоидных антител, определяются многими факторами, включая степень предшествующего йодного дефицита, величину йодной нагрузки в ходе программы йодной фортификации, генетические и экологические факторы, типичные для данной популяции. Более того, повышение уровня антител не всегда сопровождается развитием манифестного гипотиреоза, а в ряде случаев может быть и обратимым явлением [37][38].

Согласно нашим данным, первичная заболеваемость на территории Республики Беларусь тиреоидитом выросла у взрослых с 46,7 на 100 000 населения в 2009 г. до 69,6 на 100 000 населения в 2019 г. (рис. 5). Проведенный анализ показал статистически значимый рост заболеваемости указанной патологией: b=1,85 при р=0,0001; R2=0,828. Кумулятивная заболеваемость за указанный период времени составила 632,0 на 100 000 населения.

Распространенность тиреоидита за этот же период времени выросла с 543,7 до 711,8 на 100 000 населения (рис. 6). Анализ выявил статистически значимый рост распространенности указанной патологии: b=18,0 при р=0,0001; R2=0,825.

Первичная заболеваемость приобретенным гипотиреозом выросла с 42,2 на 100 000 населения в 2009 г. до 126,1 на 100 000 населения в 2019 г. (рис. 7). Был установлен статистически значимый рост заболеваемости указанной патологией: b=8,7 при р=0,0000002; R2=0,955. Кумулятивная заболеваемость за указанный период времени составила 801,93 на 100 000 населения.

Распространенность гипотиреоза за этот же период времени выросла с 336,7 до 1187,6 на 100 000 населения (рис. 8). Выявлен статистически значимый рост распространенности указанной патологии: b=87 при р=0,0000001; R2=0,961.

**Figure:**
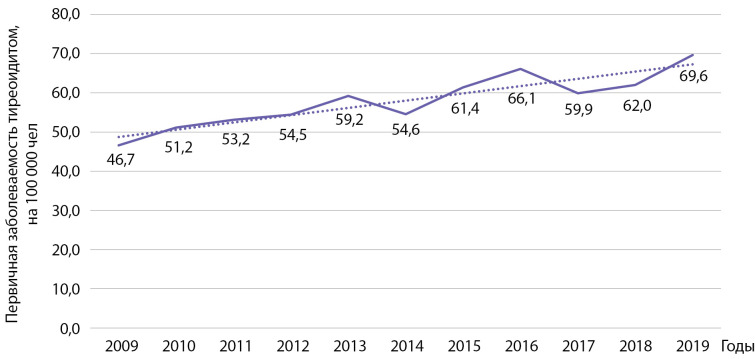
Рисунок 5. Динамика заболеваемости тиреоидитом на 100 000 человек.

**Figure:**
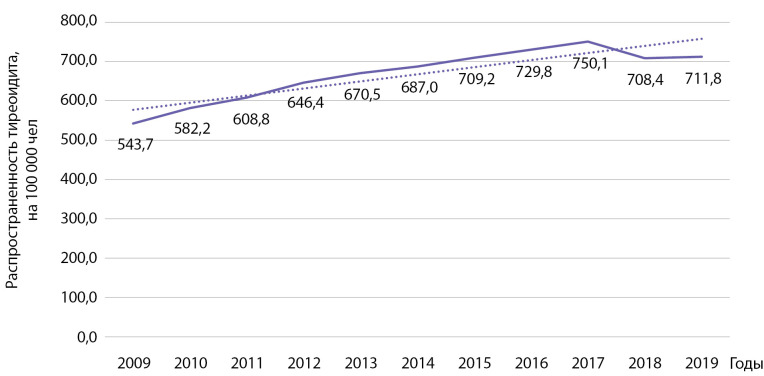
Рисунок 6. Динамика распространенности тиреоидита на 100 000 человек.

**Figure:**
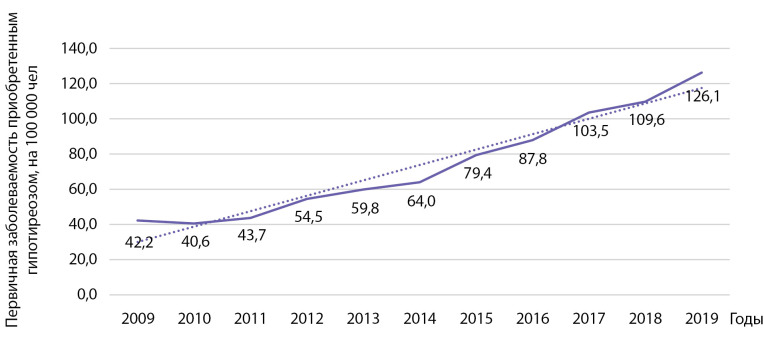
Рисунок 7. Динамика заболеваемости приобретенным гипотиреозом на 100 000 человек.

**Figure:**
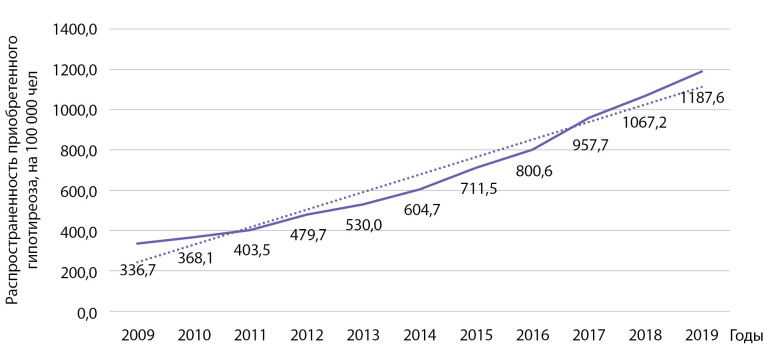
Рисунок 8. Динамика распространенности приобретенного гипотиреоза на 100 000 человек.

Сопоставление полученных нами данных с ранее опубликованными исследованиями показывает, что текущие показатели распространенности и заболеваемости населения Беларуси АИТ и приобретенным гипотиреозом соответствуют таковым для регионов с достаточной йодной обеспеченностью.

Отмечаемый достоверный рост заболеваемости и распространенности тиреоидитом и приобретенным гипотиреозом может быть связан как с увеличением доступности средств диагностики, так и с гипердиагностикой АИТ, основанной на выявлении бессимптомного носительства тиреоидных антител либо неспецифических изменений эхоструктуры ЩЖ [6], а также может объясняться отмечаемым в последние десятилетия устойчивым ростом заболеваемости аутоиммунными заболеваниями в целом, включая аутоиммунную патологию ЩЖ [38]. Проведение широкомасштабных эпидемиологических исследований с использованием стандартизированных критериев в отношении гипотиреоза и АИТ позволит уточнить истинные тенденции в изменении распространенности и заболеваемости этой патологией ЩЖ [6], выявить проблемные вопросы и обосновать актуальность их решения.

Тиреотоксикоз (гипертиреоз)

Тиреотоксикоз — синдром, обусловленный стойким избытком тиреоидных гормонов. В большинстве случаев он обусловлен гиперпродукцией гормонов ЩЖ — гипертиреозом. В соответствии с МКБ-10 тиреотоксикоз включает тиреотоксикоз с диффузным зобом (болезнь Грейвса), тиреотоксикоз с токсическим узловым зобом и другие более редкие формы.

В Европе заболеваемость тиреотоксикозом в среднем составляет 51 случай на 100 000 населения, варьируя от 38 до 127 случаев; распространенность — 750 (620–9370 случаев на 100 000 населения) [21]. В США распространенность тиреотоксикоза составляет 1200 случаев на 100 000 населения, причем 60% этих пациентов имеют манифестный тиреотоксикоз, а 40% — субклинический [39]. В России в 2018 г. распространенность тиреотоксикоза составила 132, а заболеваемость — 18 случаев на 100 000 населения [22].

Болезнь Грейвса является основной причиной тиреотоксикоза в странах с достаточным потреблением йода. Причиной ее возникновения считается развитие аутоиммунной реакции к ткани ЩЖ [39]. Токсический узловой/многоузловой зоб в большей степени характерен для населения, страдающего от хронического дефицита йода, что провоцирует рост кластеров автономно функционирующих тиреоцитов и в дальнейшем ведет к формированию многоузлового зоба с последующей его эволюцией и развитием функциональной автономии в виде многоузлового токсического зоба либо является результатом генетической мутации (токсическая аденома) [40].

При сравнительном исследовании заболеваемости тиреотоксикозом среди населения Дании с йодной недостаточностью и населения Исландии с избытком йода было установлено, что в Дании превалировал многоузловой токсический зоб (18,0 и 1,5 случая на 100 000 населения соответственно) у лиц старше 50 лет, в то время как в Исландии большинство случаев тиреотоксикоза у лиц моложе 50 лет было обусловлено болезнью Грейвса (14,8 и 19,7 случая на 100 000 населения соответственно) у лиц моложе 50 лет [41]. Сравнительное исследование 2 когорт датчан, характеризовавшихся йодным дефицитом легкой и средней тяжести соответственно, показало, что в популяции со среднетяжелым дефицитом йода стандартизированная по возрасту заболеваемость тиреотоксикозом была на 30% выше, чем у лиц, проживающих в условиях незначительного дефицита йода (60,0 и 96,7 случая на 100 000 населения/год соответственно). При этом более высокая распространенность тиреотоксикоза была обусловлена увеличением частоты многоузлового токсического зоба, в то время как распространенность болезни Грейвса была одинаковой в обеих когортах. Помимо вышеуказанного, йодный дефицит средней тяжести сопровождался более высокой заболеваемостью токсической аденомой и амиодарон-индуцированным тиреотоксикозом [42]. Последующее улучшение йодной обеспеченности позволило снизить заболеваемость до 48,8 случая на 100 000 человеко-лет даже в условиях сохраняющегося легкого дефицита йода (медианная концентрация йода в моче 74–86 мкг/л), прежде всего за счет уменьшения числа новых случаев многоузлового токсического зоба (с 44,5 до 8,2 случая на 100 000 населения/год). Заболеваемость болезнью Грейвса снизилась в меньшей степени, с 33,2 до 22,2 случая на 100 000 человеко-лет.

Сходные тенденции были выявлены и в ряде других исследований: в Швейцарии после достижения адекватной йодной обеспеченности заболеваемость узловым токсическим зобом и болезнью Грейвса снизилась и составила 9,6 и 17,4 случая на 100 000 населения/год соответственно [43], в Словении через 15 лет после достижения адекватной йодной обеспеченности заболеваемость узловым токсическим зобом и болезнью Грейвса составила 20,1 и 29,7 случая на 100 000 населения соответственно [44].

Увеличение потребления йода в популяциях с предшествующим его дефицитом в ряде случаев первоначально приводило к росту заболеваемости тиреотоксикозом, прежде всего у лиц старше 60 лет, страдающих многоузловым зобом. Это объясняется возникновением в условиях хронического йодного дефицита кластеров автономно функционирующих тиреоцитов, которые начинали гиперпродукцию тиреоидных гормонов в условиях неожиданного роста поступления йода [45]. Однако при корректном мониторинге поступления йода этот рост заболеваемости тиреотоксикозом является временным вследствие предотвращения возникновения новых автономных узлов ЩЖ в условиях достаточного поступления йода [46].

Таким образом, различия в обеспеченности йодом оказывают большое влияние на заболеваемость различными подтипами тиреотоксикоза. Незначительная и умеренная йодная недостаточность приводит к увеличению частоты узлового зоба и, как следствие, функциональной автономии ЩЖ, в особенности у лиц старших возрастных групп. Увеличение поступления йода в ранее йододефицитном регионе может приводить к транзиторному росту тиреотоксикоза за счет, опять же, функциональной автономии. Этот эффект наблюдается не всегда и может быть нивелирован постепенным увеличением потребления йода с последующим контролем количества поступающего йода. В дальнейшем происходит снижение заболеваемости тиреотоксикозом за счет снижения распространенности узлового зоба и функциональной автономии. В популяциях с достаточной либо избыточной обеспеченностью йодом ведущей причиной тиреотоксикоза является аутоиммунная патология ЩЖ, в частности, болезнь Грейвса.

На территории РБ первичная заболеваемость болезнью Грейвса выросла у взрослых с 9,7 на 100 000 населения в 2009 г. до 12,4 на 100 000 населения в 2019 г. (рис. 9). Проведенный анализ показал статистически значимый рост заболеваемости указанной патологией: b=0,374 при р=0,002; R2=0,683. Кумулятивная заболеваемость за указанный период времени составила 124,58 на 100 000 населения. При использовании полинома 6-й степени коэффициент детерминации R² составил 0,942 (р=0,0096).

Распространенность болезни Грейвса за этот же период времени выросла с 99,0 до 104,4 на 100 000 населения (b=0,865 при р=0,029; R2=0,427 (рис. 10). При использовании полинома 5-й степени коэффициент детерминации R² составил 0,904 (р=0,035).

Первичная заболеваемость другими формами тиреотоксикоза, прежде всего узловым/многоузловым токсическим зобом, выросла у взрослых с 3,7 на 100 000 населения в 2009 г. до 5,6 на 100 000 населения в 2019 г. (рис. 11). Выявлен статистически значимый рост заболеваемости указанной патологией: b=0,137 при р=0,012; R2=0,52. При использовании полинома 6-й степени коэффициент детерминации R² составил 0,994 (р=0,0001). Кумулятивная заболеваемость за указанный период времени составила 42,44 на 100 000 населения.

Распространенность узлового токсического зоба за этот же период времени уменьшилась с 34,8 до 32,2 на 100 000 населения (рис. 12). Анализ не позволил установить наличие линейного тренда: b=0,221 при р=0,121; R2=0,246.

Полученные нами данные сопоставимы с показателями распространенности и заболеваемости тиреотоксикозом в России, характеризующейся сохраняющимся йододефицитом [22], но значительно ниже европейских показателей. Обращает на себя внимание превалирование болезни Грейвса в структуре заболеваемости тиреотоксикозом, что может служить еще одним косвенным признаком, подтверждающим достаточную йодную обеспеченность населения РБ (рис. 13, 14).

**Figure:**
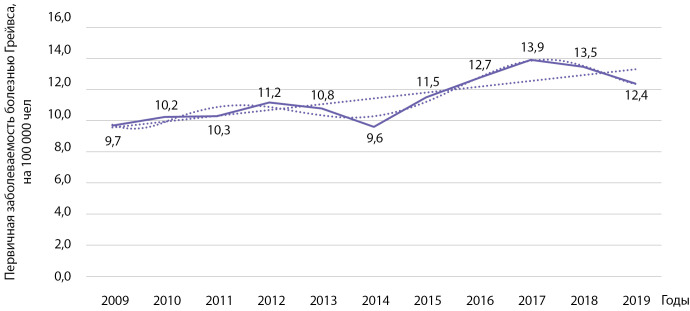
Рисунок 9. Динамика заболеваемости болезнью Грейвса на 100 000 человек.

**Figure:**
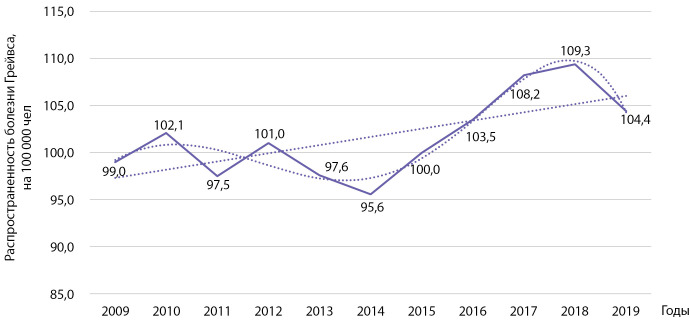
Рисунок 10. Динамика распространенности болезни Грейвса на 100 000 человек.

**Figure:**
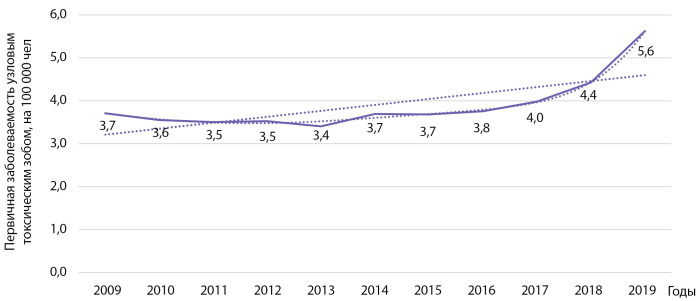
Рисунок 11. Динамика заболеваемости узловым токсическим зобом на 100 000 человек.

**Figure:**
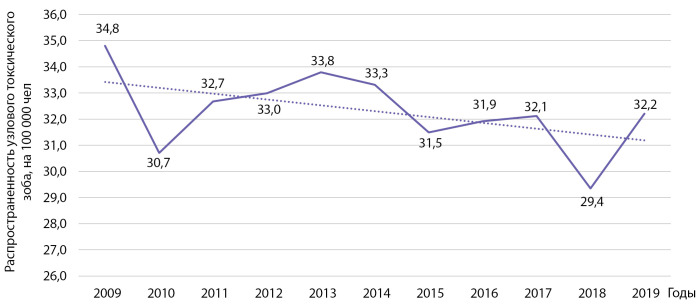
Рисунок 12. Динамика распространенности узлового токсического зоба на 100 000 человек.

**Figure:**
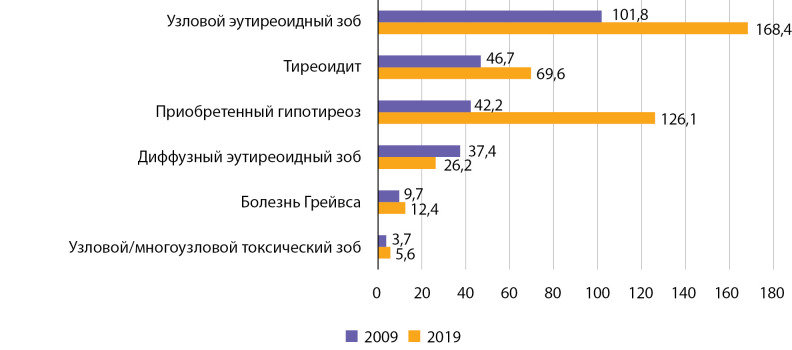
Рисунок 13. Ранжированные (по убыванию показателя в 2009 г.) показатели заболеваемости тиреоидной патологией взрослого населения РБ за 2009 и 2019 гг. (число случаев на 100 000 человек).

**Figure:**
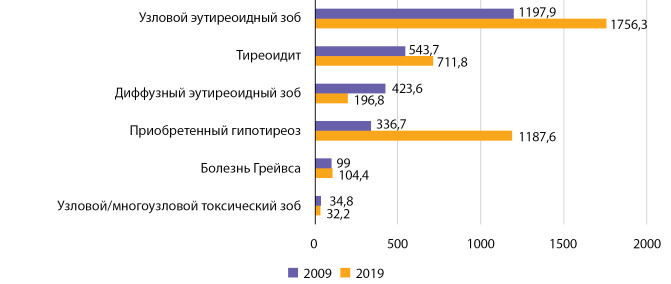
Рисунок 14. Ранжированные (по убыванию показателя в 2009 г.) показатели распространенности тиреоидной патологии у взрослого населения РБ за 2009 и 2019 гг. (число случаев на 100 000 человек).

Заболеваемость болезнью Грейвса в РБ характеризуется неустойчивой тенденцией к росту, что может объясняться как возникновением йод-индуцированной аутоиммунной патологии ЩЖ [32], так и улучшением диагностики, связанной с внедрением более чувствительных методов определения антител к рецепторам ТТГ [44]; нельзя исключать и влияние других факторов, таких как рост распространенности курения [34]. Высокие показатели коэффициента детерминации при использовании полиномиальной модели говорят о нестабильном характере роста этого показателя, который может сменяться периодами снижения заболеваемости. На наш взгляд, последнее может говорить о наличии ряда неизвестных параметров, влияющих на динамику процесса. Вместе с тем данное исследование не позволяет провести анализ причин возникновения выявленных закономерностей.

Заболеваемость узловым/многоузловым токсическим зобом также характеризуется неравномерным ростом, о чем говорит высокий коэффициент детерминации полиномиальной модели. Это может объясняться как ростом числа новых случаев тиреотоксикоза, так и совершенствованием диагностики тиреотоксикоза, в том числе его субклинических форм. На наш взгляд, последнее объяснение более вероятно с учетом того, что ранее в ряде исследований было продемонстрировано снижение заболеваемости узловым/многоузловым токсическим зобом через 15–20 лет после внедрения программ йодной фортификации, а также относительно низкими показателями заболеваемости и распространенности этой патологии у населения РБ по сравнению с данными, полученными в других исследованиях.

Отсутствие изменений в распространенности узлового токсического зоба, а также незначительный рост распространенности болезни Грейвса, на наш взгляд, можно объяснить активной хирургической тактикой, а также доступностью такой медицинской помощи населению страны.

## ЗАКЛЮЧЕНИЕ

В результате проведенных исследований были установлены снижение заболеваемости и распространенности диффузного эндемического зоба и рост данных показателей для остальных рассмотренных заболеваний ЩЖ; распространенность узлового токсического зоба не изменилась (см. рис. 13, 14).

Вместе с тем данное исследование не позволяет уточнить зависимость этих показателей от половозрастной структуры населения, йодной обеспеченности и иных факторов, а также провести анализ причин возникновения выявленных закономерностей.

На наш взгляд, это обосновывает целесообразность проведения эпидемиологических исследований, направленных на изучение структуры тиреоидной патологии у различных групп населения Республики Беларусь и выяснение истинной распространенности различных нозологических форм, поскольку большинство ранее выполненных зарубежных исследований, с результатами которых сопоставлялись наши данные, выполнялось на ограниченных когортах пациентов с применением объективных методов исследования, таких как оценка объема ЩЖ при помощи УЗИ, и единых критериев, носили сплошной проспективный характер; все это значительно повышало их точность и надежность.

Требуют дальнейшего изучения причины увеличения заболеваемости тиреоидитом и гипотиреозом, исключение гипердиагностики этих состояний.

Выявленная тенденция к росту заболеваемости узловым/многоузловым эутиреоидным зобом обусловливает необходимость выяснения причин возникновения этой тенденции, а также разработки и внедрения новых высокоточных методов предоперационной диагностики морфологической природы этих новообразований щитовидной железы, что в условиях постоянно увеличивающегося их количества позволит избежать множества необоснованных операций и высвободить резервы для оперативных вмешательств у тех пациентов, кто в этом нуждается.

Узловой/многоузловой токсический зоб представляет собой серьезную медико-социальную проблему вследствие своей распространенности преимущественно у лиц старших возрастных групп. Неуклонный рост заболеваемости этой патологией требует внедрения в клиническую практику малоинвазивных и органосберегающих методов лечения, позволяющих ликвидировать тиреотоксикоз с наименьшим количеством побочных эффектов.

## ДОПОЛНИТЕЛЬНАЯ ИНФОРМАЦИЯ

Источники финансирования. Исследование выполнено в рамках научно-исследовательской работы (НИР) «Разработка новых методов диагностики и лечения хирургических болезней органов брюшной и грудной полостей, эндокринной и сосудистой патологии» №20190619 (сроки выполнения 2019–2023 гг.).

Конфликт интересов. Салко Ольга Борисовна — главный внештатный специалист по эндокринологии Министерства здравоохранения Республики Беларусь, заместитель главного врача по организационно-методической работе Государственного учреждения «Республиканский центр медицинской реабилитации и бальнеолечения».

Остальные авторы декларируют отсутствие явных и потенциальных конфликтов интересов, связанных с публикацией настоящей статьи.

Участие авторов. Якубовский С.В. — концепция и дизайн исследования, сбор и обработка материалов, анализ полученных данных, написание текста; Кондратенко Г.Г. — концепция и дизайн исследования, редактирование текста; Салко О.Б. — анализ полученных данных, редактирование текста; Кузьменкова Е.И. — концепция и дизайн исследования, анализ полученных данных. Все авторы одобрили финальную версию статьи перед публикацией, выразили согласие нести ответственность за все аспекты работы, подразумевающую надлежащее изучение и решение вопросов, связанных с точностью или добросовестностью любой части работы.

Благодарности. Мы благодарим Государственное учреждение «Республиканский научно-практический центр медицинских технологий, информатизации, управления и экономики здравоохранения», отдельно руководителя отдела медицинской статистики и мониторинга здоровья населения Атрашкевич Терезу Ивановну за предоставленные статистические данные и помощь в их обработке.
